# Microseismicity and lithosphere thickness at a nearly-amagmatic oceanic detachment fault system

**DOI:** 10.1038/s41467-023-36169-w

**Published:** 2023-01-26

**Authors:** Jie Chen, Wayne C. Crawford, Mathilde Cannat

**Affiliations:** Université Paris Cité, Institut de physique du globe de Paris, CNRS, F-75005 Paris, France

**Keywords:** Seismology, Tectonics, Geophysics

## Abstract

Oceanic detachment faults play a central role in accommodating the plate divergence at slow-ultraslow spreading mid-ocean ridges. Successive flip-flop detachment faults in a nearly-amagmatic region of the ultraslow spreading Southwest Indian Ridge (SWIR) at 64°30’E accommodate ~100% of plate divergence, with mostly ultramafic smooth seafloor. Here we present microseismicity data, recorded by ocean bottom seismometers, showing that the axial brittle lithosphere is on the order of 15 km thick under the nearly-amagmatic smooth seafloor, which is no thicker than under nearby volcanic seafloor or at more magmatic SWIR detachment systems. Our data reveal that microearthquakes with normal focal mechanisms are colocated with seismically-imaged damage zones of the active detachment fault and of antithetic hanging-wall faults. The level of the hanging-wall seismicity is significantly higher than that documented at more magmatic detachments of slow-ultraslow ridges, which may be a unique feature of nearly-amagmatic flip-flop detachment systems.

## Abstract (web summary)

Oceanic detachment faults play a central role in accommodating the plate divergence at mid-oceanic ridges. Here, the authors show micro-seismicity of a nearly-amagmatic flip-flop detachment fault system at the ultraslow spreading Southwest Indian Ridge.

## Introduction

**Fig. 1:**
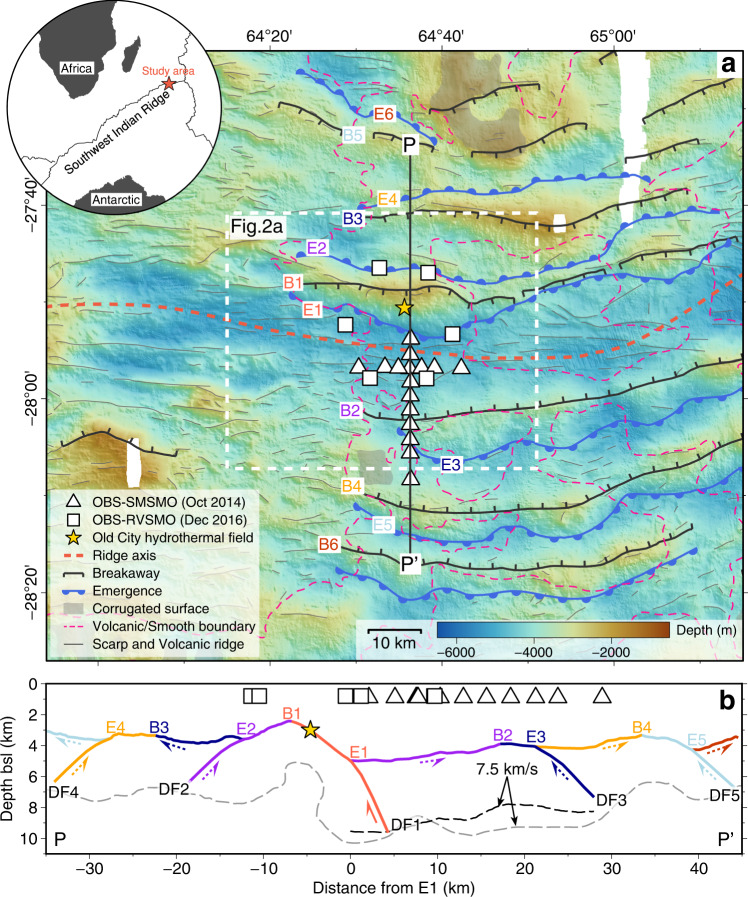
OBS locations and tectonic interpretation of the flip-flop detachment fault system at the eastern SWIR. **a** Tectonic map with locations of the OBS networks: SMSMO (14 OBSs) and RVSMO (6 OBSs). The base map is created based on shipborne bathymetry data^4^. Geological and tectonic information (see legend for symbols) includes breakaways (B1–B6) and emergences (E1–E6) of successive detachment faults, corrugated surface, the boundary between volcanic and smooth seafloor, and linear sketches of scarps and volcanic ridges^1^. Yellow star is the low-temperature Old City hydrothermal field with carbonate-brucite chimneys^5^. The white dashed square marks the bounds of Fig. 2a. **b** Tectonic interpretation of the successive flip-flop detachment fault system along the PP’ cross-section^1^. Black and gray dashed lines are the 7.5 km/s velocity contours constrained by seismic tomography^23,39^. Locations of OBSs are projected to the PP’ cross-section. bsl below sea level, SWIR Southwest Indian Ridge, DF detachment fault, OBS ocean-bottom seismometer, SMSMO SISMOSMOOTH cruise, RVSMO ROVSMOOTH cruise.

The flip-flop detachment fault system discovered at a nearly amagmatic region of the ultraslow spreading Southwest Indian Ridge (SWIR; full spreading rate of 14 mm/yr) near 64°30’E represents a previously unknown seafloor spreading mode^1–3^ (Fig. 1a). In this mode, detachment faults accommodate nearly 100% of plate divergence, continuously cutting into the footwalls of their predecessors, with flipping polarities every 0.6–1.5 Ma^1,2^ (Fig. 1). The resulting seafloor morphology is the so-called smooth seafloor^4^, with extensive exposure of mantle-derived peridotites, only patches of hummocky basalts^2^, and low-temperature carbonate-brucite hydrothermal chimneys^5^. This newly discovered seafloor spreading mode differs from the “classic” detachment-volcanic and volcanic–volcanic modes at slow spreading ridges and at more magmatically robust portions of ultraslow spreading ridges, where at least one plate is dominated by abyssal-hill volcanic seafloor^4,6–9^ and the detachment fault is characterized by dome-shaped corrugated surface^10–12^.

**Fig. 2:**
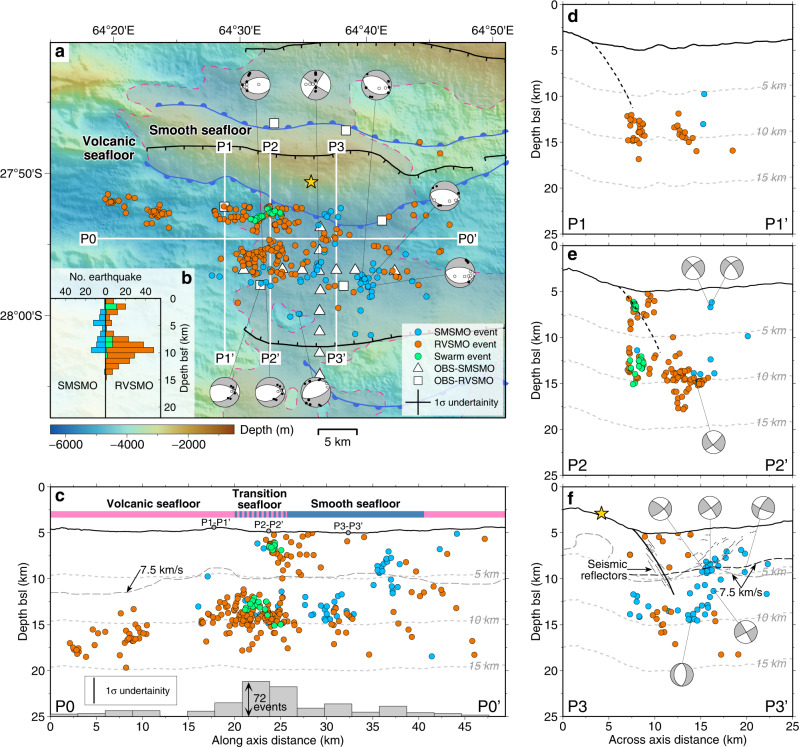
Distribution of earthquakes and focal mechanisms. **a** Bathymetric map of the SWIR 64°30’E area showing 307 events of the SMSMO (blue circles) and RVSMO (orange circles) catalogs, the RVSMO seismic swarm (green circles), and 8 determined focal mechanisms. Cross in the legend shows the average absolute horizontal location uncertainty of 3.2 km (1*σ*). Geological information (see legend for symbols) includes breakaways, emergences, and the boundary between volcanic and smooth seafloor^1^. Best-fitting focal mechanisms have clear upward (black dots) and downward (white dots) first motions of P-wave onsets (see the “Methods” section). Depth profiles (P0–P0’ to P3–P3’) are marked as labeled white lines in **a**. **b** Histograms of earthquake depths below the seafloor for the SMSMO (blue) and RVSMO (orange) catalogs, including the seismic swarm (green). **c** Along-axis depth profile P0–P0’ projecting earthquakes within ±8 km off the profile (VE = 1 and the same below). The vertical bar shows the average absolute vertical uncertainty of 2.8 km (1*σ*). The classification of volcanic (pink) and smooth (blue) seafloor is indicated^4^. Gray dashed line is the 7.5 km/s velocity contour^39^. Labeled gray dashed lines show depths below the seafloor (the same below). **d** and **e** Across-axis depth profiles P1–P1’ and P2–P2’ projecting earthquakes within ±3 km off the profiles. Black dashed lines indicate the fault plane adapted from (**f**). **f** Across-axis depth profile P3–P3’ projecting earthquakes within ±5 km off the profile. Dotted lines represent seismic reflectors, interpreted as DF1 damage zone^23^, with the main detachment fault plane tentatively drawn in the center (subseafloor solid black line). Black^23^ and gray^39^ dashed lines are the 7.5 km/s velocity contours. Focal mechanisms are projected to profiles P2–P2’ and P3–P3’.

Seismicity provides a means to study magmatic, tectonic, and hydrothermal processes within the lithosphere of mid-ocean ridges^13–19^ (MORs) and is an indirect proxy for the thermal regime by constraining the depth to the base of the brittle lithosphere^20,21^. Here, we present a catalog of 307 microearthquakes recorded during two short (8 and 19 days for the SMSMO and RVSMO catalogs, respectively) ocean bottom seismometer (OBS) deployments (Figs. 1, 2a; see the “Methods” section). These microearthquakes and 8 focal mechanisms (see the “Methods section) reveal a unique distribution of geodynamic stress and accommodation at the youngest active detachment system (DF1) of the SWIR 64°30’E.

## Results and discussion

### Microseismicity at the SWIR 64°30’E

The two short OBS deployments offer snapshots in time of the seismic activity at the youngest active detachment system. The average seismicity rate is 11.4 events per day (8.4 and 12.7 events per day in the SMSMO and RVSMO catalogs, respectively), including a 34-event seismic swarm in the RVSMO catalog, during December 22–23, 2016 (Supplementary Fig. 5). Most microearthquakes occurred in the axial valley (Fig. 2a) between the emergence of DF1 (E1) and the breakaway of DF2 (B2). The highest number of events is recorded near the P2–P2’ cross-axis profile (Fig. 2a, c). Earthquake hypocenters have depths between 0 and 15 km below the seafloor (bsf), and events in the shorter SMSMO catalog were mostly at <10 km bsf (Fig. 2b). Local magnitudes (*M*_L_) range from −0.5 to 3.2, with a magnitude of completeness of 1.1 and a *b*-value of 0.9 based on the Gutenberg–Richter relation^22^ (see the “Methods” section and Supplementary Fig. 6). Focal mechanisms (see the “Methods” section) correspond to normal faulting, as expected in an extensional context, except for one strike-slip faulting event (Fig. 2a).

Many microearthquake hypocenters plotted in the cross-axis profiles P1–P1’ and P2–P2’ (Fig. 2d, e) are aligned with the trace of the subseafloor detachment fault plane as inferred from a series of subparallel seismic reflectors with a dip of 50–60°^23,24^. In all three cross-axis profiles, and particularly P3–P3’, several events are scattered in the detachment hanging wall. Focal mechanisms of these hanging-wall earthquakes display a prevalence of normal faults with an average dip of 50° at 2–7 km bsf (Fig. 2e, f). These dips are consistent with the geometry of nearby north-dipping seismic reflectors^23^ (Fig. 2f) and with small-offset fault scarps at the seafloor^2^, suggesting that these small faults are conjugate with the detachment fault.

Along the ridge axis (P0–P0’), we also observed a larger number of earthquakes at the transition between volcanic and smooth seafloor (near the P2–P2’ profile; Fig. 2c, e). Combining this observation with the previous observation of subhorizontal seismic reflectors interpreted as intrusive magmatic sills beneath this transition^24^, we propose that seismicity may be elevated here because the crystallized basalts or gabbros (beneath the volcanic seafloor) are more prone to seismogenic rupture than the ultramafic basement (beneath the smooth seafloor) in which stress may be accommodated by creep due to serpentinization. Our observations may thus provide a framework to examine earthquake generation at detachment systems, interacting with sparse magmatism.

### Maximum depth of earthquakes and the axial thermal regime

The thickness of the axial brittle lithosphere, which is commonly interpreted by the maximum depth of earthquakes^20,25,26^, is predicted to increase as the spreading rate decreases^20^ and/or as melt supply decreases^27^. The 64°30’E region of the ultraslow spreading SWIR, being nearly amagmatic, may be regarded as a calibration for the brittle lithosphere thickness of the MOR system. Although our two OBS deployments only recorded a few hundred microearthquakes, the maximum depth of earthquake hypocenters is on the order of 15 km, and this depth is mostly uniform along the ridge axis, across the transition from volcanic to nearly avolcanic smooth seafloor (Fig. 2c). The thickness of the brittle lithosphere is mainly controlled by the thermal regime with its base roughly corresponding to the ~650 °C isotherm^21,28,29^. This temperature condition is consistent with the minimum depth of 18 km for the 800–1000 °C isotherm at the SWIR 64°30’E, determined from petrological constraints for the high-stress ductile deformation of sheared peridotites^30,31^.

A greater maximum depth of microearthquakes (~20 km) was found at the adjacent magma-poor SWIR 65°10’E^32^, but these earthquakes were located using a significantly different velocity model constrained from the magmatically robust segment #8 volcano^33^ (Supplementary Fig. 2). We also obtain a maximum earthquake depth of ~20 km if we apply their velocity model to our events (see the “Methods” section and Supplementary Fig. 8), although this model is absolutely unsuitable to the nearly amagmatic setting of the SWIR 64°30’E.

Intriguingly, the maximum earthquake depth at the Dragon Horn area (SWIR 49°36’E), which has the same spreading rate but is more magmatic than the SWIR 64°30’E, is also on the order of 15 km^18,34^. There thus appears to be a disconnection between the maximum earthquake depth and melt supply at ultraslow spreading ridges, putting into question a direct relationship between the thickness of the axial brittle lithosphere and the amount of melt supply to the ridge axis. A recent numerical thermal model provides a possible explanation by considering melt emplacement depth^35^: at a given spreading rate and melt supply, the thermal regime can be colder if melt is emplaced in the shallow hydrothermally active region. This shallow melt body will be cooled very rapidly, leading to an axial thermal regime beneath the hydrothermal domain that is identical to no melt supply^35^.

### Microseismicity at detachment systems

**Fig. 3:**
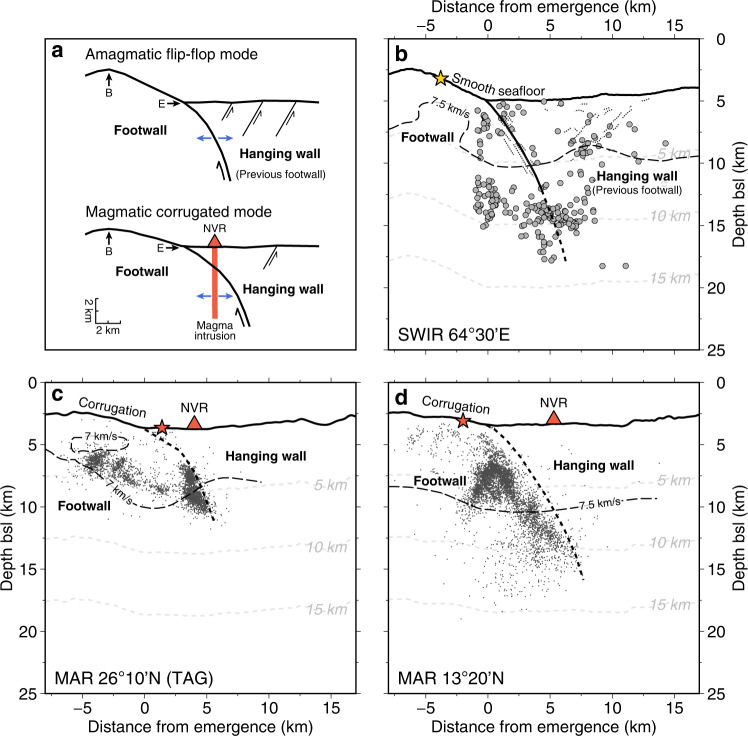
Modes of detachment faults and their patterns of seismicity. **a** 2D conceptual sketch of amagmatic flip–flop and magmatic corrugated modes of detachment faults. The flip-flop mode may have more hanging-wall faults to accommodate part of the plate spreading, while this part of plate spreading at the corrugated mode is accommodated by magma intrusions. **b**–**d** Microseismicity at the SWIR 64°30’E (this study), and the MAR 26°10’N^15^ and 13°20’N^16,17^ (VE = 1). Labeled dashed black lines show velocity contours of 7.5 km/s (SWIR 64°30’E^39^ and MAR 13°20’N^53^) and 7 km/s (MAR 26°10’N^54^). Yellow star is the low-temperature Old City hydrothermal vent. Red stars are high-temperature hydrothermal vents. MAR Mid-Atlantic Ridge, NVR neovolcanic ridge, B breakaway, E emergence.

The seismicity pattern of the active flip-flop detachment fault at the SWIR 64°30’E is characterized by earthquakes aligned with the trace of the detachment fault plane and by coinciding with antithetic normal faults in the detachment hanging wall (Fig. 3b). The detachment-faulting events are similar to those observed at the more magmatic corrugated detachment faults, such as at 26°10’N^15^ (TAG), 13°20’N^16,17^, and 14°50’N (Logatchev) of the Mid-Atlantic Ridge^15–17,19^ (MAR) and at the SWIR Dragon Horn^18,34^. However, these detachment faults have very few hanging-wall earthquakes (Fig. 3). We propose that the hanging-wall seismicity may be a signature of the nearly amagmatic flip–flop detachment system, where the plate spreading is accommodated by both detachment and hanging-wall faulting with very few magma intrusions. In contrast to the corrugated mode of detachment faults, magma intrusions are responsible for a considerable part of the plate spreading in the hanging wall, which largely reduces the hanging-wall faulting and seismicity.

In conclusion, our study of two short OBS deployments at an active flip-flop detachment fault system in the nearly amagmatic 64°30’E section of the SWIR reveals a maximum depth of earthquakes of 15 km and hanging-wall seismicity not observed at more magmatic mid-ocean ridge detachment systems. It also suggests that there is a disconnect between the maximum earthquake depth and melt supply at ultraslow spreading ridges.

## Methods

### Microearthquake experiments

The first experiment, using 14 OBSs in a cross configuration, recorded 8 days of microearthquakes in between airgun shots during the SISMOSMOOTH active seismic survey in October 2014 (R/V *Marion Dufresne*; SMSMO catalog; Fig. 1). The second experiment, using 6 OBSs in a hexagon configuration, continuously recorded 19 days of microearthquakes during the ROVSMOOTH cruise in December 2016 (R/V *Pourquoi Pas?*; RVSMO catalog; Fig. 1). Each OBS recorded three orthogonal ground motions plus pressure signals, with all channels recording at 250 Hz during the SISMOSMOOTH cruise and at 500 Hz during the ROVSMOOTH cruise.

### Earthquake detection

The internal clocks of the OBSs were synchronized on deployment and recovery, and a linear drift correction was applied. Earthquake events were network detected by the CONDET program in the SEISAN software^36^ using the STA/LTA trigger algorithm, and an automatic picking procedure^37^ was used to pick P and S wave arrival onsets. These events were registered in the SEISAN database, and P- and S-wave arrival onsets were manually refined (Supplementary Fig. 1).

### 1-D velocity model

The 1-D P-wave velocity model was calculated using the VELEST program^38^. The initial velocity model was extracted from a seismic refraction experiment across DF1 from the SISMOSMOOTH cruise^23^. This model generally agrees with a broader velocity model at the same area^39^ (Supplementary Fig. 2). Only events with OBS ≥ 6 and GAP ≤ 180° were used in the VELEST program. The best-fitting P-wave velocity model was iteratively searched (Supplementary Fig. 2). The final root-mean-square (RMS) is 107 ms. The S-wave velocity (Vs) model is calculated using a best-fitting Vp/Vs ratio of 1.7, based on the Wadati diagram that plots the travel time of P-wave versus travel time differences of P- and S-waves (S–P time; Supplementary Fig. 3).

### Earthquake location and relocation

The initial earthquake locations were searched by the NonLinLoc software with the Oct-tree algorithm^40^ and the SWIR 64°30’E velocity model (Supplementary Fig. 2). The maximum-likelihood hypocenter is used to locate each earthquake event, and a three-dimension error ellipsoid (68% confidence) is generated from the posterior density function (PDF) scatter samples^40^. 507 events of SMSMO (122) and RVSMO (385) catalogs were located with four or more stations, and 388 events of SMSMO (88) and RVSMO (300) catalogs are well located with horizontal and depth errors of <5 km and RMS residual of <100 ms (Supplementary Fig. 8a-1–d-1). Station corrections, given by the average travel time residuals at each OBS calculated by the NonLinLoc software in an iterative way, were applied for the P and S phases (Supplementary Fig. 4): the mean absolute station correction is 60 ± 40 ms in both catalogs. P- and S-waves travel time residuals follow the Gaussian distribution with an average RMS misfit of 34 ms (Supplementary Fig. 9). Bootstrap analysis of location errors was applied for four chosen groups of NonLinLoc located events to show the stability of the hypocenter estimates: (1) two swarm events in the RVSMO catalog, (2) two deep events in the RVSMO catalog (beneath smooth and volcanic seafloor), (3) one hanging-wall event in the SMSMO catalog, and (4) two detachment-fault events in both SMSMO and RVSMO catalogs (Supplementary Fig. 10).

We also applied the SWIR 65–66°E velocity model^32^ (Supplementary Fig. 2), constrained from a more magmatically robust area than our study area^23,33^, to locate earthquakes recorded in our study area using the NonLinLoc software (Supplementary Fig. 8a-2–d-2). Earthquakes with more distant epicenters to the OBS network tend to have deeper hypocenters to form an inverted V shape of along-axis hypocenter depth distribution (Supplementary Fig. 8b-2), which is similar to what was proposed at the SWIR 65-66°E^32^.

NonLinLoc hypocenters with six or more stations, horizontal and depth errors of <5 km, and RMS residual of <100 ms, were relocated using the Double-Difference Hypocenter (HypoDD) algorithm^41^. The relocation uses both catalog and cross-correlation (Supplementary Fig. 11a-3–d-3) and runs using the python module HypoDDpy^42^. A time window of 300 ms was applied based on pickings of P and S arrival onsets, and cross-correlated waveforms with a correlation coefficient <0.6 were rejected. Supplementary Fig. 12 shows the differences between the NonLinLoc locations and the HypoDD relocations for 30 randomly selected events. 307 well‐constrained absolute hypocenters were relocated with mean relative location errors of 500 m E–W, 400 m N–S, and 500 m in depth.

We also tested catalog only (Supplementary Fig. 11a-1–d-1) and cross-correlation only (Supplementary Figs. 11a-2–d-2) in the HypoDD algorithm, which show more located earthquakes than both catalog and cross-correlation (Supplementary Fig. 11a-3–d-3). Hypocenter location patterns in the three algorithms are relatively stable.

### Earthquake magnitude calculation

The definition of local magnitudes (*M*_L_) is given by^43^

1 $${{M}}_{{{{{{\rm{L}}}}}}}=\log \left({A}\right)+n\,\log (r)+K\,r+{C},$$

where *A* (in nm) is the maximum amplitude of horizontal components picked in the Wood–Anderson seismogram^44^, *r* (in km) is the hypocentral distance, *C* is a correction for each OBS, and *n* and *K* are constants to be calculated, and related to geometrical spreading and attenuation of seismic waves, respectively. The local magnitudes, parameters *n* and *K*, and station correction can be solved by a least-squares criterion that produces an optimal solution^45,46^. For the SMSMO and RVSMO catalogs together, we obtain *n* = −2.923, *K* = 5.85 × 10^−3^, and *C* = (−3)−(−2). Magnitude completeness (*M*_c_) is determined as 1.1 using the *b*-value stability approach^47^ and resulting in a *b*-value of 0.9.

### First-motion focal mechanism

To calculate best-fitting focal mechanisms, we use two first‐motion-based algorithms, HASH^48^ and FOCMEC^49^. Multiple criteria were applied: apparent first-motion polarities of P-wave onsets ≥8, azimuthal gaps ≤250°, the weighted fraction of misfit polarities <10%, RMS of fault plane uncertainty from HASH ≤ 35° (95% confidence), and similar reasonable solutions generated by both approaches. Eight acceptable focal mechanisms were found in the SMSMO catalog (Fig. 2). The azimuthal gaps of accepted focal mechanisms are all >90°, so their quality grades based on the HASH criteria are all in category E (*A* is the best constrained). We did not obtain focal mechanisms in the RVSMO deployment due to the lesser number of OBSs.

## Supplementary information

Supplementary Information File

Peer Review File

Supplementary Data

## Data Availability

OBS locations, two earthquake catalogs, swarm events, and focal mechanisms in this study are provided in the Supplementary Information. Any remaining raw datasets of this study and additional information on the SISMOSMOOTH^50^ and ROVSMOOTH^51^ cruises are available at 10.17600/14003300 and 10.17600/16002000.

## Code availability

The software and codes used in this study included: SEISAN software^36^ (http://www.seisan.info/), VELEST program^38^ (https://seg.ethz.ch/software/velest.html), NonLinLoc software^40^ (http://alomax.free.fr/nlloc/), Double-Difference Hypocenter (HypoDD) algorithm^41^ (Version 2.0; https://www.ldeo.columbia.edu/~felixw/hypoDD.html) and its python module HypoDDpy^42^ (10.5281/zenodo.18907), HASH^48^ and FOCMEC^49^ programs internally in the SEISAN software, and GMT^52^ (Version 6; https://www.generic-mapping-tools.org/).
