# Supplementary material for: Microseismicity and lithosphere thickness at a nearly-amagmatic oceanic detachment fault system
Source: Nat Commun. 2023 Jan 26;14:430. doi: 10.1038/s41467-023-36169-w (PMC9879945; doi:10.1038/s41467-023-36169-w)
Supplement: Supplementary file 1 — Supplementary Information File [file 41467_2023_36169_MOESM1_ESM.pdf]

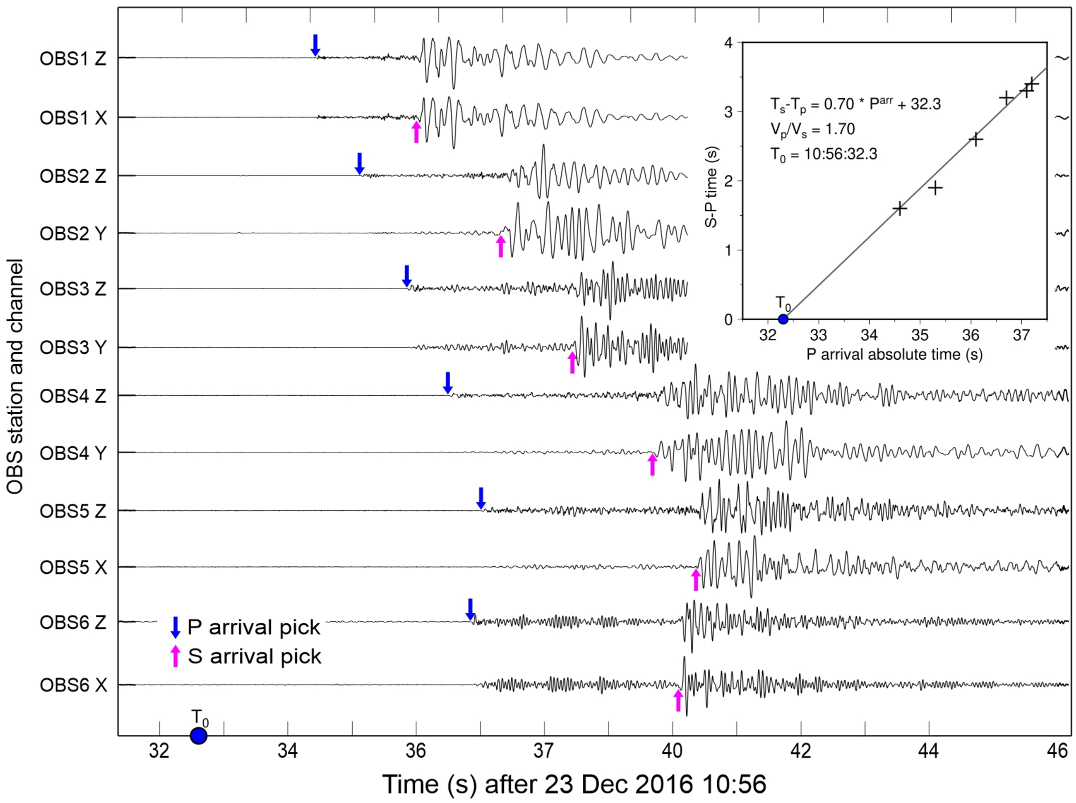

Supplementary Fig. 1 | Typical event waveforms. The event occurred at 64.394°E/27.883°S 11.7 km below seafloor with a local magnitude of 2.3, at 10:56, 23 Dec 2016 in RVSMO catalog. The inset shows the P-wave arrival absolute time versus arrival time differences of P- and S-waves (S-P time), yielding the origin time (T0) of 10:56:32.3 and a Vp/Vs ratio of 1.7.

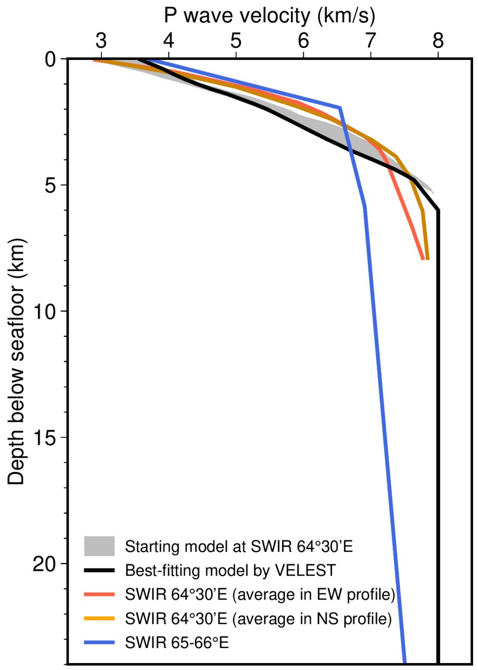

Supplementary Fig. 2 | 1-D P wave velocity model. Starting model (gray area) at the SWIR 64°30'E is extracted from a seismic refraction experiment[23]. The best-fitting model (black line) is iteratively searched by the VELEST program[38]. Red and orange lines are average velocity models in EW and NS profiles at the SWIR 64°30'E[39]. Blue line is the velocity model at the SWIR 65-66°E[32].

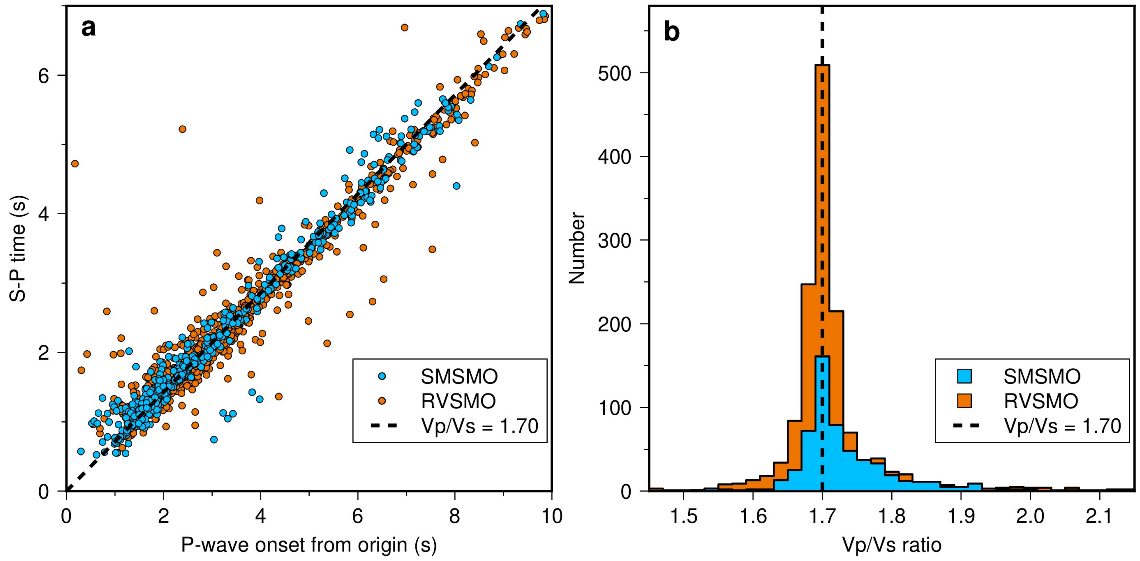

Supplementary Fig. 3 | Wadati diagram. (**a**) P-wave onset from origin versus S-P time (Wadati diagram) for both SMSMO (orange) and RVSMO (blue) catalogs. (**b**) Histogram of Vp/Vs ratio Vp/Vs ratio is calculated from the slopes of the Wadati diagram. The best-fitting Vp/Vs ratio is 1.7.

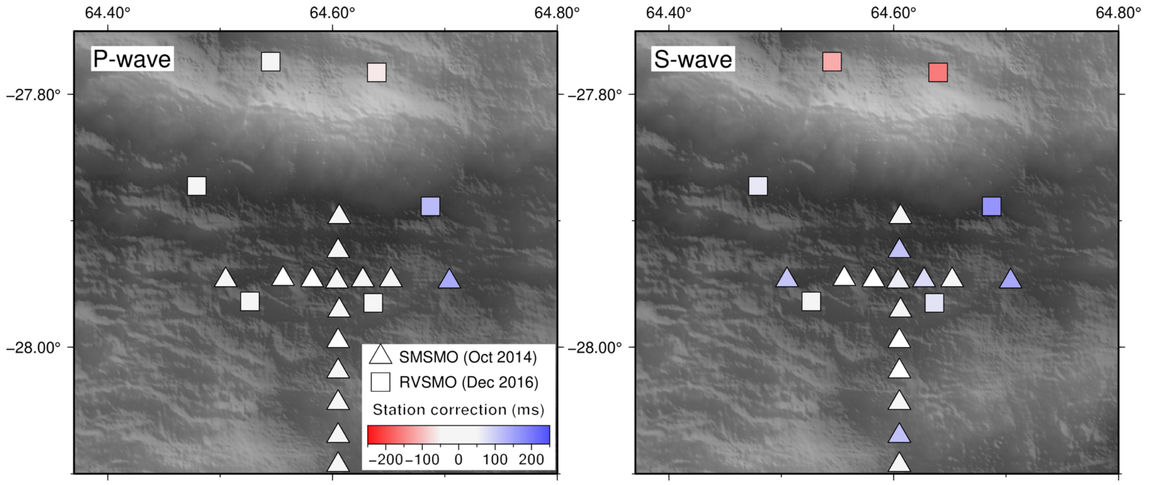

Supplementary Fig. 4 | Station corrections of P- and S-wave returned by NonLinLoc.

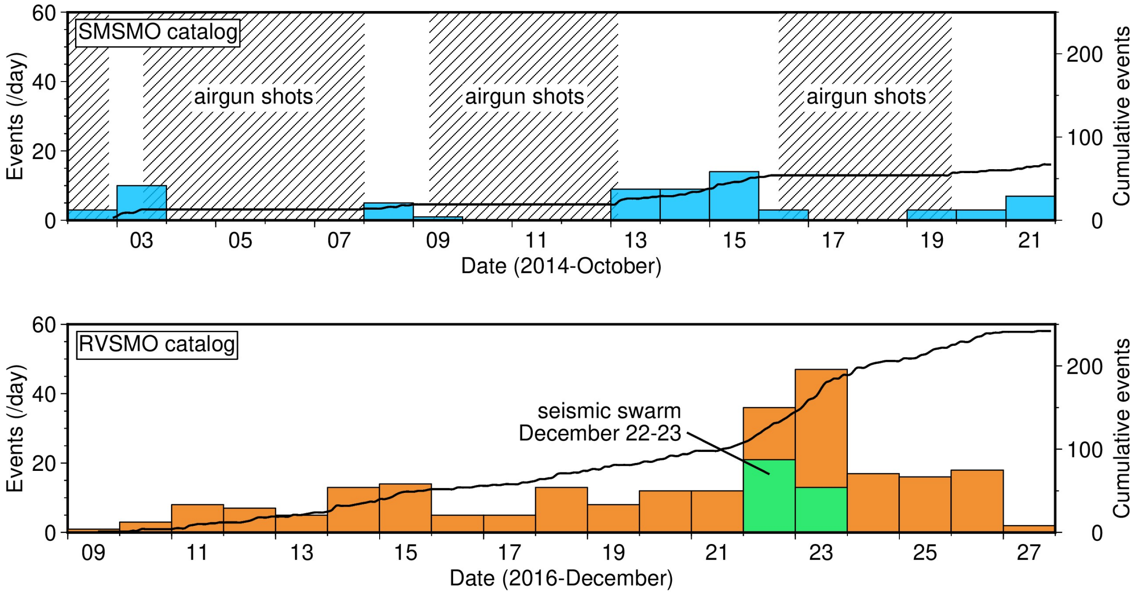

Supplementary Fig. 5 | Histogram and cumulative histogram of located earthquakes for the SMSMO and RVSMO catalogs.

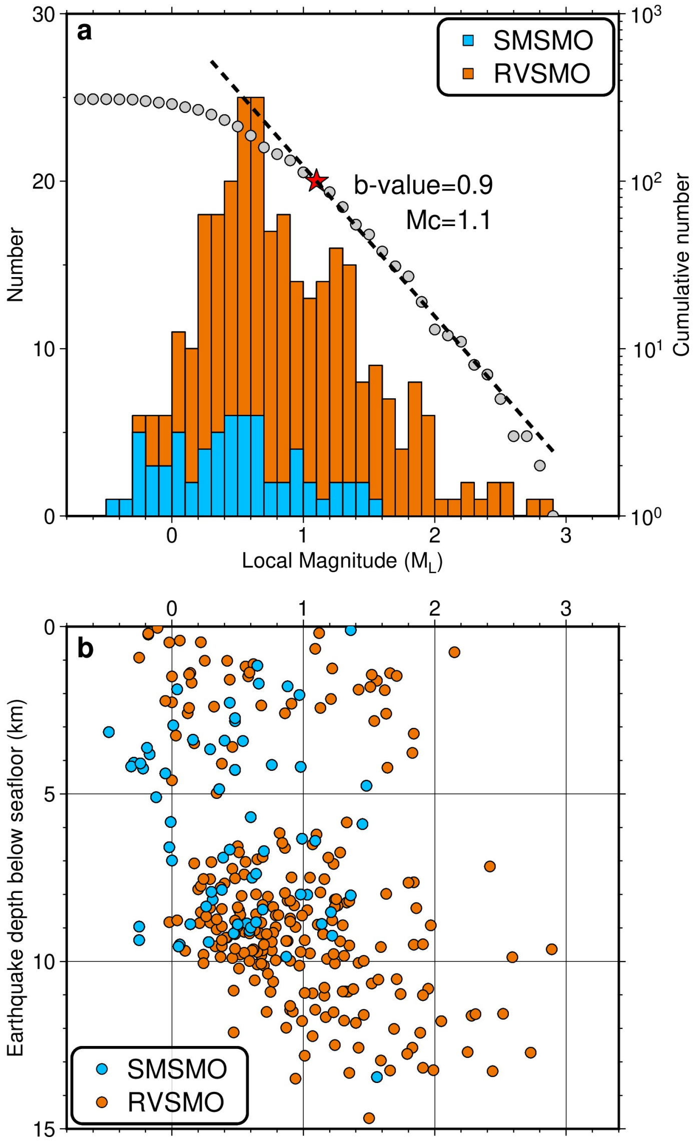

Supplementary Fig. 6 | Distribution of local magnitude (M_L). (**a**) Histograms of local magnitude for the SMSMO (blue) and RVSMO (orange) catalogs (left Y-axis). The cumulative number of earthquakes for both catalogs (gray circles) as a function of local magnitude (right Y-axis). Magnitude completeness (Mc) is determined as 1.1 (red star) using the b-value stability approach[47], resulting in a b-value of 0.9 (slope of the dashed line). (**b**) Local magnitude as a function of earthquake depth for the SMSMO (blue) and RVSMO (orange) catalogs.

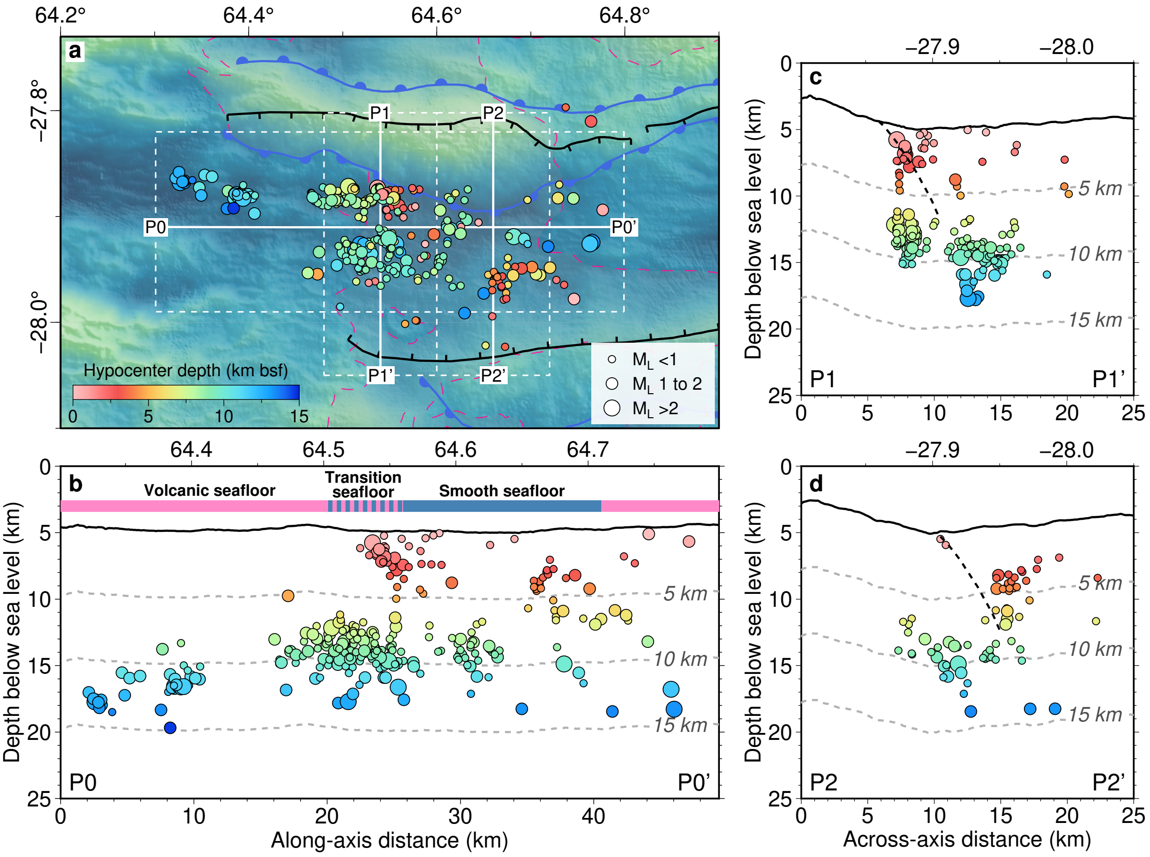

Supplementary Fig. 7 | Earthquake relocations, color coded based on hypocenter depths and circle size based on local magnitude (M_L).

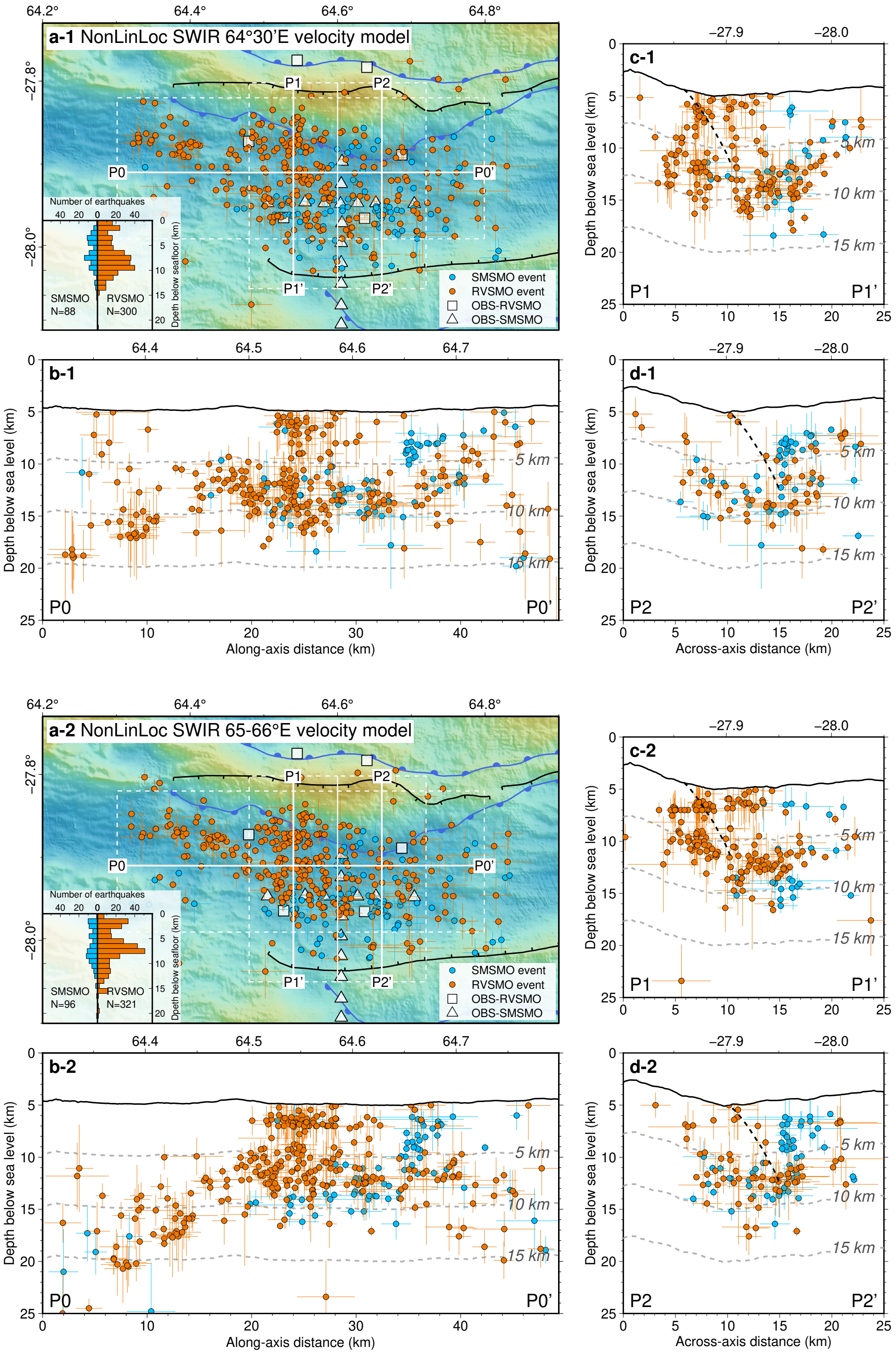

Supplementary Fig. 8 | Earthquake locations. See legend for symbols. Earthquake locations using the NonLinLoc with the SWIR 64°30'E velocity model (**a-1** to **d-1**). Earthquake locations using the NonLinLoc with the SWIR 65-66°E velocity model (**a-2** to **d-2**), compared with the NonLinLoc results with the SWIR 64°30'E velocity model (pale gray). Velocity models see Supplementary Fig. 2. Error bars represent absolute location errors.

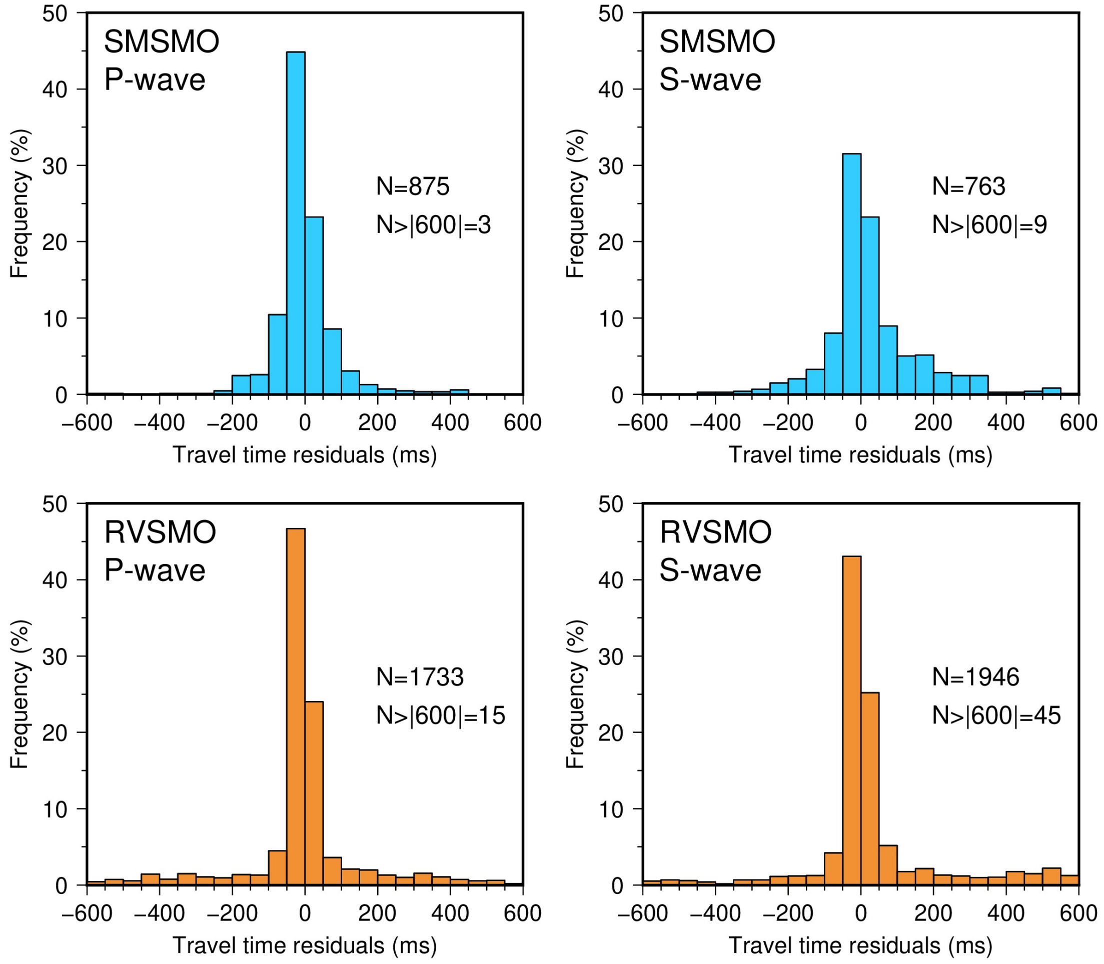

Supplementary Fig. 9 | Frequency distribution of P- and S-wave travel time residuals for the SMSMO (blue) and RVSMO (orange) catalogs.

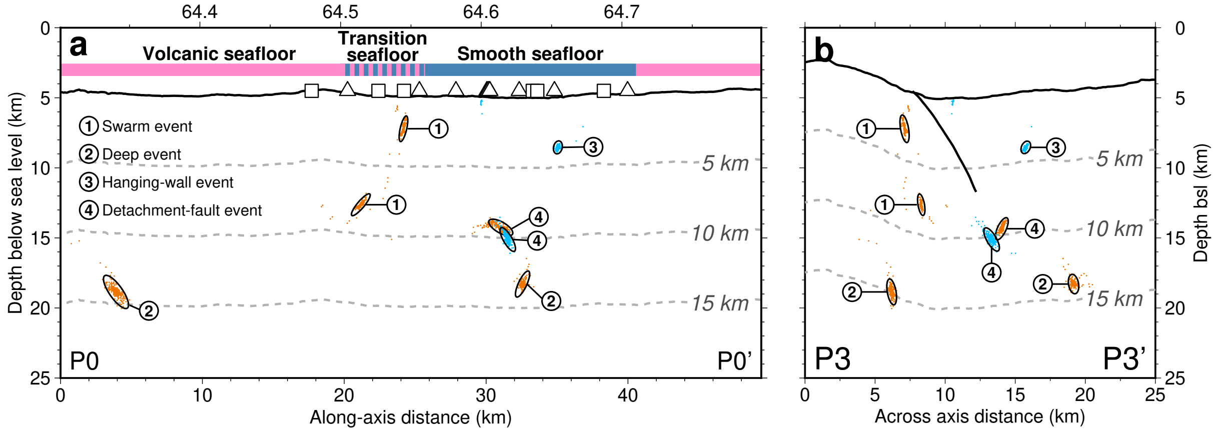

Supplementary Fig. 10 | Bootstrap analysis of location errors for four chosen groups of NonLinLoc located events. Blue and orange dots represent events in the SMSMO and RVSMO catalogs, respectively. Perturbations were randomly applied 300 times to P and S arrivals, which were constrained by travel time residuals shown in Supplementary Fig. 9.

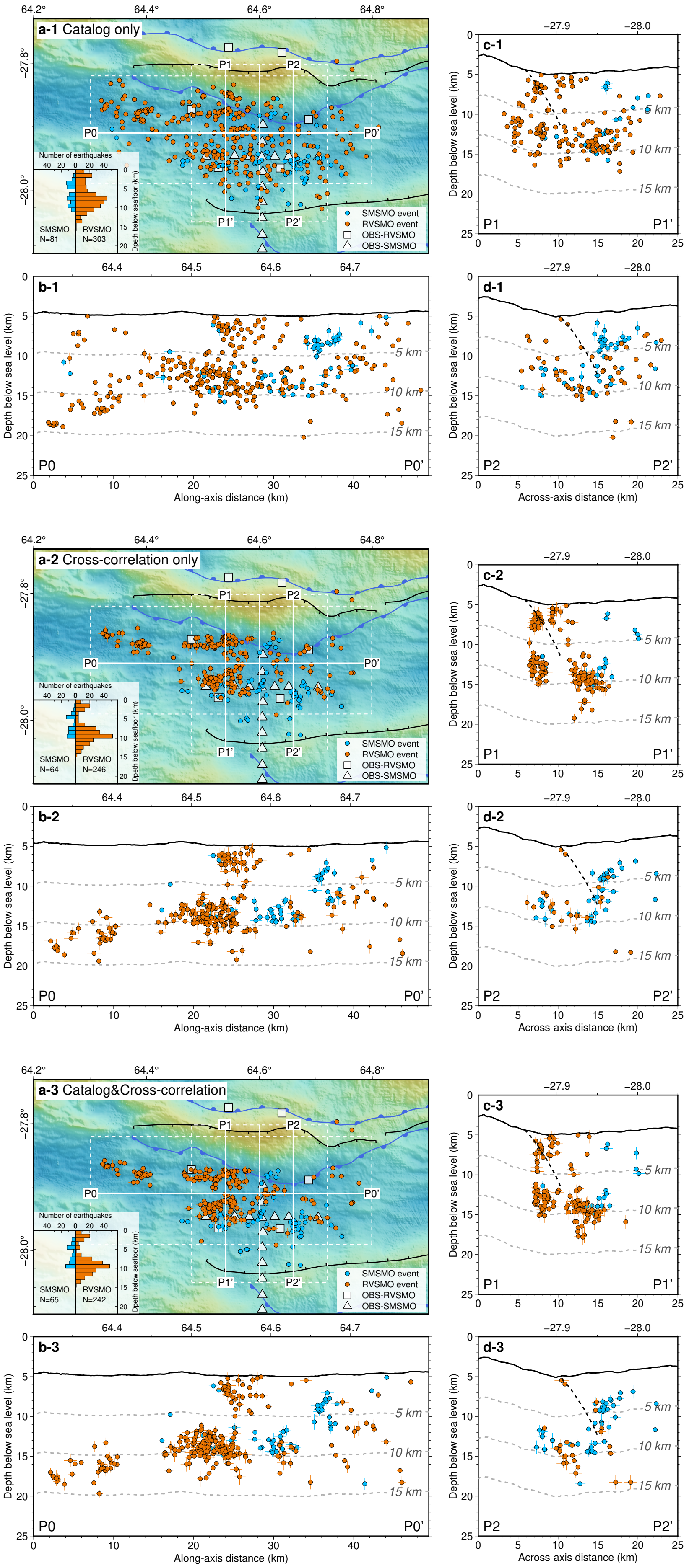

a-1 Catalog only
a-2 Cross-correlation only
a-3 Catalog&Cross-correlation
64.2°
64.4°
64.6°
64.8°
-27.8°
-28.0°
P0
P0'
P1
P1'
P2
P2'
Number of earthquakes
Depth below seafloor (km)
SMSMO N=81
RVSMO N=303
SMSMO N=64
RVSMO N=246
SMSMO N=65
RVSMO N=242
SMSMO event
RVSMO event
OBS-RVSMO
OBS-SMSMO
b-1
b-2
b-3
c-1
c-2
c-3
d-1
d-2
d-3
Depth below sea level (km)
Along-axis distance (km)
Across-axis distance (km)
5 km
10 km
15 km
64.4
64.5
64.6
64.7
-27.9
-28.0

Supplementary Fig. 11 | Earthquake relocations. See legend for symbols. Tests of earthquake relocation using the HypoDD with catalog only (**a-1** to **d-1**), cross-correlation only (**a-2** to **d-2**), and catalog&cross-correlation (**a-3** to **d-3**; same results as Fig. 2). Error bars represent relative location errors.

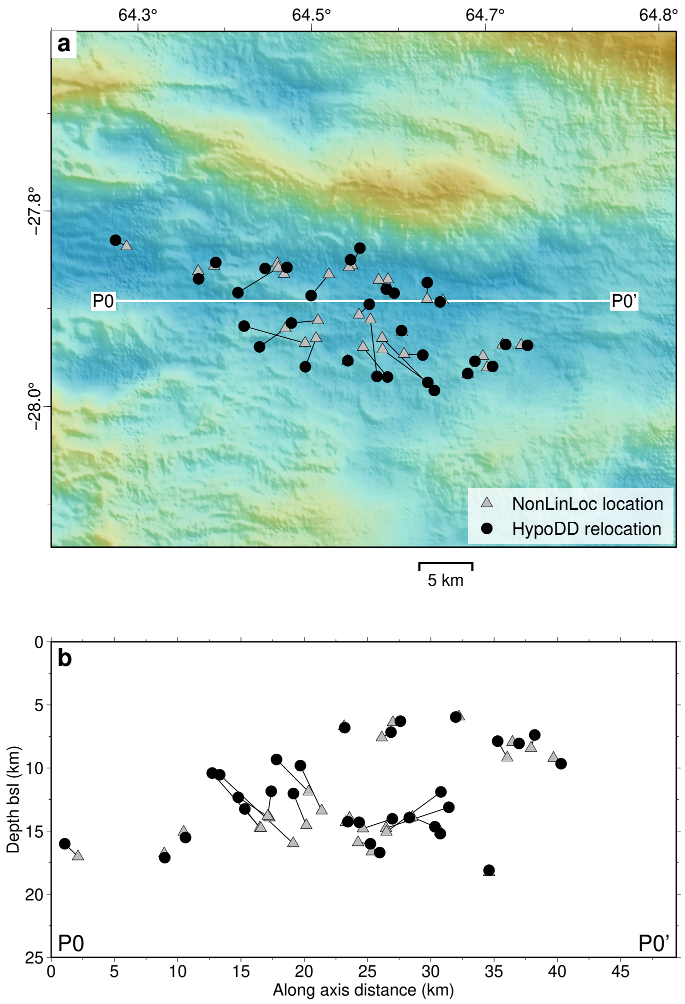

Supplementary Fig. 12 | Differences of NonLinLoc locations and HypoDD relocations for 30 randomly selected events. Each event is connected by a black line between the results of the NonLinLoc location and the HypoDD relocation (catalog&cross-correlation). (**a**) Map view. (**b**) events projected on the along-axis P0-P0' profile.
