# Supplementary material for: Microseismicity and lithosphere thickness at a nearly-amagmatic oceanic detachment fault system
Source: Nat Commun. 2023 Jan 26;14:430. doi: 10.1038/s41467-023-36169-w (PMC9879945; doi:10.1038/s41467-023-36169-w)
Supplement: Supplementary file 2 — Peer Review File [file 41467_2023_36169_MOESM2_ESM.pdf]

nature portfolio

Peer Review File

Microseismicity and lithosphere thickness at a flip-flop detachment fault system of a nearly-amagmatic mid-ocean ridge

Open Access This file is licensed under a Creative Commons Attribution 4.0 International License, which permits use, sharing, adaptation, distribution and reproduction in any medium or format, as long as you give appropriate credit to the original author(s) and the source, provide a link to the Creative Commons license, and indicate if changes were made. In the cases where the authors are anonymous, such as is the case for the reports of anonymous peer reviewers, author attribution should be to 'Anonymous Referee' followed by a clear attribution to the source work. The images or other third party material in this file are included in the article's Creative Commons license, unless indicated otherwise in a credit line to the material. If material is not included in the article's Creative Commons license and your intended use is not permitted by statutory regulation or exceeds the permitted use, you will need to obtain permission directly from the copyright holder. To view a copy of this license, visit http://creativecommons.org/licenses/by/4.0/.

Peer Review Comments file

Reviewer comments, first round review:

Reviewer #1 (Remarks to the Author):

1) What are the noteworthy results?

This manuscript, although based on a fairly short passive seismic survey as byproduct of an active seismic survey gives an impression of the earthquake activity of an active detachment at a magma-starved portion of an ultraslow spreading ridge. Interestingly, the seismic activity does not happen mostly in the footwall of the detachment but also or even to a greater extent in the hanging wall of the detachment. Since passive seismic surveys are still very rare at ultraslow spreading ridges, this is a noteworthy result that helps to accumulate observations on how deformation and extension takes place at the slowest spreading mid-ocean ridges.

2) Will the work be of significance to the field and related fields? How does it compare to the established literature?

As mentioned above, this work contributes valuable information to a still growing body of observations that will help to understand how mid-ocean ridges produce new lithosphere in the absence of robust melt supply. The manuscript discusses all existent literature on seismicity surveys of mid-ocean ridge detachment faults and places the new observations of a seismically active hanging wall in the context of existing observations. The manuscript therefore also constitutes a fine overview of the current knowledge on detachment seismicity.

3) Does the work support the conclusions and claims, or is additional evidence needed?

The time period of the seismic survey is rather short, but sufficient to get a good estimate of for example the maximum depth of faulting used to draw conclusions on the thermal regime. However, when it comes to interpreting the temporal behaviour of the seismicity, the time period may be a little short to assess the role of the swarm activity. The three clusters observed each consist only of very few events. It would be helpful to see, whether seismicity in these spatial clusters more frequently happens in temporal clusters of few events and whether the deep and shallow spatial clusters are usually linked. While the interpretation of an intrusion event is certainly justified and appears obvious, similarly located seismicity clusters (relative to the detachment) in the study of Parnell-Turner et al. 2017 also show step-like increases in the cumulative number of earthquakes, but these clusters were mainly considered to happen in an area of compression due to bending of the footwall. Since the evidence of a migration of hypocenters is somewhat limited in the present study, it might make sense to discuss alternative explanations or mark the interpretation of swarm seismicity as intrusion more clearly as speculative.

4) Are there any flaws in the data analysis, interpretation and conclusions? Do these prohibit publication or require revision?

There are no flaws in the data analysis, interpretation and conclusions. The manuscript is well structured, clearly written and the argumentation is sound.

5) Is the methodology sound? Does the work meet the expected standards in your field?

The methodology is state-of-the-art in seismology and meets the expected standards. Some of the figures, in particular 2 and 3 can be improved by adding additional information to facilitate a 3-D impression of the detachment seismicity and earthquake magnitudes.

6) Is there enough detail provided in the methods for the work to be reproduced?

I recommend to expand the method section slightly, adding more information on the quality of the earthquake locations. How were reliably located earthquakes selected? Could double-difference relocation be used to establish a migration of hypocenters? The sensitivity of the hypocenter solutions with respect to the choice of a velocity model is nicely demonstrated. However, the location quality of individual earthquakes should not be solely based on RMS values. Since this information is all available from the output of the location algorithm, including this additional information only requires minor revision.

In conclusion, I consider this manuscript a helpful contribution to the study of ultraslow mid-ocean ridge spreading processes in an area that provides significant extra information for a comprehensive understanding of these processes. The manuscript is well written and requires only minor revision, improving figures, detailing location quality and revisiting the interpretation of the seismic swarm activity. I attach an annotated manuscript with more detailed comments.

Vera Schlindwein

Reviewer #2 (Remarks to the Author):

Review of "Microseismicity and lithosphere thickness at a nearly magmatic mid-ocean ridge"

by Jie Chen, Crawford, and Cannat

Review prepared by Robert Sohn, Woods Hole Oceanographic Institution

The authors present results from two ocean bottom seismometer (OBS) deployments at the Southwest Indian Ridge (SWIR) at 64°30'E. On the basis of their hypocentral analyses, they have three primary results: (1) 15-km thick seismogenic lithosphere, which they interpret as challenging current models of mid-ocean ridge (MOR) thermal regimes, (2) unusually large amounts of seismicity in the hanging wall, which they say 'provide a framework to examine earthquake generation at detachment systems, interacting with sparse magmatism', and (3) a two-day swarm that is interpreted to represent a magmatic event, possibly coupled with tectonic spreading.

Before going into a detailed review of this work, it is important to provide some context. Compared to subaerial volcanic and tectonic systems, the amount of seismicity data we have for submarine volcanic and tectonic systems is very small. This is due to obvious technological reasons, but it is worth remembering, especially since the majority of the Earth's volcanic and fault systems are located in submarine environments. The limited seismicity data we do have from the global MOR system tends to preferentially come from certain parts of the global ocean that are relatively easy to access from North American and European ports. It just so happens that the ultra-slow spreading ridges are located in hard-to-access parts of the global ocean. The slowest of them all, the Gakkel Ridge, is under the Arctic pack ice. The next slowest, the SWIR, is in the 'Roaring Forties' in the Indian Ocean, a remote location with notoriously rough seas. Until very recently we essentially had no local microearthquake data from the SWIR, and little-to-none from the Gakkel.

This means that the data presented here are valuable because they are rare. Moreover, much of the microearthquake data that has been published for the SWIR has been of low quality due to a lack of rigor in the analytical techniques. This unfortunate situation has led to a great deal of confusion regarding the deformation mechanisms at ultra-slow ridges, including the depth extent of seismicity, which is an important topic that is covered in this manuscript. Through the Extended Data and Supplementary information provided in the Methods section I was able to verify that the earthquake hypocenters, and results, more generally, were generated via a rigorous analytical procedure. This provides a level of confidence in the results presented in this manuscript that is much stronger than that associated with most previous microearthquake studies at the SWIR.

The primary drawback to this manuscript is that the OBS deployments from which the results are drawn were very short - 8 and 19 days, respectively. To put this in perspective, we can think

about the characteristic time scales associated with tectono-magamatic cycles at MORs, which in a general sense are a function of spreading rate. At the study site for this work, spreading is accommodated via 'flip flop' detachments (which is quite interesting in and of itself), such that the characteristic time scale is roughly that of the life cycle of an individual detachment, which, according to previous work, is 0.6-1.5 Ma. In total, the OBS deployments thus covered an infinitesimally small fraction of the tectono-magmatic cycle (2.7 x 10^-5 %). Any interpretation of these data must keep this important caveat in mind. A secondary drawback is that the OBS networks that were deployed are not optimal for microearthquake analyses - for RVSMO there are too few instruments, and for SVSMO the instruments are deployed on lines for generating 2-D tomography models from airgun shots.

To evaluate the manuscript, I assess the three primary results individually.

(1) Lithospheric thickness. The authors find a maximum depth of seismicity, and thus an estimated lithospheric thickness, of 15 km. As mentioned previously, their analytical techniques are sound, and this result is robust. Moreover, it is consistent with petrological constraints. This puts an important new point on the global dataset of depth of seismicity vs. spreading rate compilation, since we have precious few points at the ultra-slow end of the spectrum. They then compare this value against the 18 km max. depth of seismicity published for the Dragon Flag detachment system at a nearby segment of the SWIR that is more magnetically robust. They state that this ~3 km depth discrepancy 'questions current numerical models of … the thermal regime of the MOR', and suggest this may be due to hydrothermal heat removal. Neither of the two papers that have been published regarding seismicity at the Dragon Flag area provide enough information to assess the quality of the hypocenter estimates (unlike this manuscript), even though one of them was published in the longer format Journal of Geophysical Res. The ~3 km difference in maximum depth of seismicity is well within the 'noise' given the depth uncertainties for this type of work, especially given the lack of information in the previous two papers, and I don't think it is interpretable - certainly not a large enough difference to merit questioning MOR thermal models.

Comment #1 - There has been enough controversy regarding the depth of seismicity at the SWIR to warrant a careful treatment of this topic. This may be why the authors devote text, both in the main part of the manuscript and in the methods and Supplementary materials, describing other microearthquake studies from the SWIR and the methods used therein. However, I found this aspect of the manuscript somewhat confusing. I suggest the authors address this topic in a more straightforward way, by saying, for example, something like - this is the max. depth of seismicity from our study, and differences between our results and other published results are likely due to differences in the velocity models and methods used to estimate hypocenters. If it is necessary to provide more detail than this, then it is probably also necessary to publish in a longer-format journal where it is possible to go into all the details.

Comment #2 - This manuscript has the opportunity to 'right a wrong' that it doesn't quite take. The anomalously large depths of seismicity published in reference #32 have been debunked, but this was done in a somewhat obscure way, buried in a Geology manuscript that many readers of Nature and associated publications will not see. As a result, many non-specialists still believe that earthquakes at the SWIR extend to depths > 30 km, which is unfortunate. This paper has the opportunity to set the record straight, but it doesn't quite do that. It could say that these new results, from possibly the most magmatic (and thus coldest) section of the SWIR, support the Grevemeyer et al. 2019 results showing that lithospheric thickness at ultra-slow ridges is on the order of 15, and not 30 km. It would be appropriate to do that in a Nature journal as a way of correcting an erroneous result previously published in Nature.

(2) Hanging wall seismicity. The authors find that a significant amount of the detected seismicity occurs in the hanging wall of the active detachment system, and state that this differs from seismicity at other detachments, where the hanging wall is largely inactive. This is indeed interesting, and it is true that in previous experiments hanging wall seismicity was not generally observed. One of the interesting things about this study that may be somewhat undersold in the manuscript is that it is the first such study at a site of 'flip flop' detachments, where essentially 100% of spreading is accommodated tectonically. Perhaps hanging wall seismicity is a feature of such sites? It would make sense. With a longer deployment and a more optimal seismic network it

might be possible to assign these events to individual faults, which would be really interesting.

(3) Two-day swarm. The authors detected 34 earthquakes during a 2-day period of the RVSMO deployment. Overall, the events have an east-west spread, with a deeper cluster and a shallow cluster. They interpret this as a magmatic, or mixed magmatic-tectonic swarm based on: 1) upward migration of activity, 2) location beneath volcanic seafloor. I did not find this interpretation convincing. The evidence for upward migration is weak, and the east-west spread of the activity confounds a straightforward magmatic interpretation. The total number of events (34) is very small for a magmatic event, especially considering that 12 of them have a tectonic interpretation. A b-value for this swarm is not presented, but the lack of a mainshock-aftershock pattern appears to be the strongest evidence for a magmatic origin. However, in the existing literature I don't recall evidence for mainshock-aftershock patterns at detachment faults. Rather, the fault systems seem to generate fairly constant seismicity. This, combined with the fact that the swarm events fall within the same area as non-swarm events detected by the network, suggest to me that the swarm was simply a period of slightly elevated seismicity rates. The statements 'The nature of the deep-east cluster is not clear, but is expected for a melt injection in this extensional context.', and 'This could mean that melt intruding into the brittle peridotite at depth altered the stresses in the detachment footwall, so that a shallow portion of the fault which was near to rupture broke.', are too vague. Finally, the mantra, as I understand it, is that magmatic events at fast spreading ridges are frequent and small, whereas at ultra-slow spreading ridges they are infrequent and large. My expectation would thus be that any magmatic events at this magma-starved area would be large in volume and temporal extent, which is inconsistent with the data presented here. I think the authors have a fairly high bar to clear for assigning a magmatic origin to any of the observed seismic events, and they have not done that in my opinion. I think this aspect is a distraction from the much more robust and more important result that the maximum depth of seismicity at one of the most magmatic segments of the global MOR is ~15 km.

Review Summary:

The authors present new data and results from OBS deployments on the SWIR. In particular, their results come from a segment where extension is accommodated nearly 100% by tectonic processes - i.e., flip-flop detachments. To my knowledge this is the first such experiment at such a site. As a result, the data are valuable and the results are noteworthy. The primary limitation of the manuscript is that the OBS deployments, and thus the data acquisition periods, are very short. A secondary limitation is that the OBS networks are not optimal for microearthquake studies, which leads to relatively large hypocentral errors in some cases, and a general inability to obtain reliable focal mechanisms. The methods used to analyze the seismic data and estimate hypocenters are well-described and sound. I trust the results. The lithospheric thickness estimate derived from the hypocenters is robust and believable. The fact that the maximum depth of seismicity at one of the most magmatic ridge segments in the global MOR system is ~15 km (not 30+ km) is an important observation that needs to be disseminated to the community. The observation of hanging wall seismicity is novel for oceanic detachment settings, and may have to do with the unique tectonic setting. This is an interesting result that could be expanded upon in terms of relating it to flip-flop detachments. I was not convinced by the authors' interpretation of the 34-event swarm as being of magmatic origin, and I recommend removing this from the manuscript as it is too speculative and not supported by sufficient evidence.

Comments on specific parts of the manuscript:

Line 54-55: I think you need to be careful talking about a seismic gap based on data from such short (8 and 19 days) deployments. Since this gap isn't discussed any further in the manuscript, it's probably better to simply delete this statement.

Regarding the density of seismicity - for each deployment, the highest levels of seismicity were detected near the geometric center of the networks. This isn't surprising, and indicates that detectability is exerting a strong influence on the spatial hypocentral patterns.

Lines 89-93: Grevemeyer et al., 2019 showed that the anomalously large depths for the

Schlindwein and Schmid study were the result of not properly accounting for thick sediments beneath the OBSs. I suggest saying precisely that instead of "using a velocity model derived from…". Please see my comments regarding depth of seismicity in other parts of this review

Lines 94-95: The maximum depth of earthquakes at the TAG segment in the deMartin et al., 2007 paper is 10 km, not 6-7 km.

Lines 98-99: 'Dragon Flag' or 'Dragon Horn'? In the Tao et al paper it is Dragon Horn. Unfortunately, the Tao et al paper provides very little information regarding the details of the microearthquake analysis, which makes those results hard to assess. I don't think, though, that those results provide cause to question current numerical models.

Lines 102-105: As noted above, I don't think the results to-date provide a reason to question our understanding of the links between spreading rate, melt supply, and thermal regime. These last two sentences in this paragraph should be deleted. They are too speculative.

Figure 1: I suggest expanding the latitude extent of this figure, especially to the south. It would be nice to have a view of the entire across-axis section for understanding the local tectonics.

Figure 2: Panel 2a is a key figure for the paper. It is too busy. There is too much information cluttered into a small area. Suggestions: 1) remove the dashed squares. The relevant length scales for the profile boundaries can be simply stated in the text. 2) remove the colored dashed lines for volcanic/smooth seafloor, or make them thinner at the very least. If I'm seeing correctly, the smooth seafloor regions have some kind of opacity mask on them. Perhaps that is enough for this figure? 3) The white lines for the profiles are layered on top of the earthquakes and everything else - move them down below the earthquakes. 4) Remove the labels for the breakaways and emergences since they are already identified in Figure 1. 5) The legend in the lower right corner infringes too much on the map. One possibility would be to move panel (b) out of the frame and move the legend to the lower left corner. Or the latitude bounds of the map could be increased to provide a bit more separation between the legend and the 'active' part of the map.

Panel 2c: It probably isn't necessary to draw the cross-section line identifiers through the depth section. Putting a 'P1', 'P2', and 'P3' marker at the seafloor interface for each section should be enough.

Panel 2d:

Hypocentral errors: The nominal errors for the hypocentral parameter estimates need to be discussed somewhere - probably in the Methods document. I could not find a discussion of the error bars in the manuscript or the Methods. The location uncertainties for many of these events are going to be large, particularly for deep events with nearly vertical take-off angles from the source, and for events that are outside, or on the fringes, of the network apertures. Uncertainties for all of the RVSMO events will be relatively large due to the small number of instruments.

The most important interpretations in this manuscript pertain to event depths. The depth uncertainties therefore must be discussed in some detail. Although the confidence intervals for the uncertainties/error bars in Figures 2c-f are not specified, the error bars are almost certainly too small. To take an extreme example, the depth error bars for the events at the west edge of profile P0 are on the order of 1 km, despite the fact that the events are well outside the network aperture, and have depths greater than ~15 km. The depth constraints on these events will be very weak, and certainly greater than the plotted error bars.

This is a small detail, but in the various figures throughout the manuscript error bars should not extend above the seafloor interface.

Is it possible that the error bars represent relative, as opposed to absolute, errors?

Figure 3: This figure is shown to support the assertion of a swarm that migrates spatially in time. The figure does a nice job of showing the relationship between time and event position, but I must

say I don’t interpret it the same way as the authors. Specifically, I don’t see evidence for vertical migration in time, as indicated by the upward pointing arrow in panel (c). In that panel, I see two clusters of seismicity (deep vs shallow), and the deep cluster seems to originate at a depth of~12.5 km, and then spread both up and down. Indeed, both the shallowest and deepest events in this cluster occur at the end of the swarm.

The situation is complicated by a distinct east-to-west spread in the activity, which is contemporaneous. I’m not sure what exactly to make out of this swarm, but I do think that: (1) the evidence for any kind of systematic upward migration is weak, (2) the swarm events are located within the same regions as many non-swarm events - none of which are attributed to magmatism/diking, (3) 34 earthquakes over 2 days would be a very small number for a magnetically triggered swarm, especially in low-magma-supply terrain where these events should release a considerable amount of tectonic strain. I am thus skeptical of the interpretation of these events as being related to a magmatic event.

Methods: I found the Earthquake location and relocation section confusing. In particular, lines 319-331 appear to describe methods used in other studies, rather than this one. I’m not sure that is appropriate. This section describes how different velocity models were used to estimate hypocenter, which is fine, but in a short-format journal I think it is better to simply describe results from the ‘best’ velocity model. You can say you tried other velocity models, but providing too much detail about that process may be confusing to the reader.

Extended data, Figure 6: In panel (b), there should be a general trend between earthquake size and depth (deeper = larger), and indeed there is. However, the very largest event (ML > 3) is at a depth of ~0 km, which is almost certainly in error. This event, and the next largest event from the SMSMO deployment should be examined as they location/size estimates are probably incorrect. In general, events that are both large and shallow should be examined for potential errors.

Supplementary Figures: I appreciate the authors including these figures. In general, they provide a lot of information that a specialist (such as myself) can use to assess their results, which is, unfortunately, not typical. It looks like using the ’65-66°E’ velocity model gets rid of most of the large/shallow events, which is likely more accurate. I don’t think, however, it is necessary to include the gray dots in the second set of panels since those events can be seen in the first set of panels, and they add clutter to an already busy figure.

Reviewer comments, second round review:

Reviewer #2 (Remarks to the Author):

Review prepared by Robert Sohn, Woods Hole Oceanographic Institution

Review Summary: In this revised manuscript the authors describe results from an OBS microearthquake study at the SWIR 64°30'E - a site of flip-flop detachment faulting. To my knowledge, this is the first OBS seismicity experiment at a site of flip-flop detachments and smooth seafloor, such that this is the community's 'first look' at active faulting in this type of environment. The data are thus unique, and the results should be published. The authors have chosen to focus, though, on the depth of seismicity, and to use this to argue that the 'classic' relationship between seismicity depth and spreading rate does not hold for ultra-slow spreading ridges. I have two issues with this: 1) I don't think we can properly assess the relationship between earthquake depth and thermal environment without first having an understanding of how the flip-flop detachment environment influences seismicity and deformation, and 2) The manuscript only brushes the surface of the topic, and does not adequately account for uncertainties, both in the presented hypocenters, and in differences between OBS experiments conducted at different sites.

In my assessment the manuscript is thus not yet ready for publication. If the authors wish to keep the current emphasis on implications for thermal environment, a more thorough analysis is required, as I describe, below. I don't know if it is possible to adequately do this within the space constraints of Nature Comms & Environment. My recommendation, however, would be to focus on the seismicity of flip-flop detachment environments, as this is the truly unique aspect of the results. The authors have the opportunity to make the first assessment of how the somewhat bizarre mechanics of flip-flop detachment faulting affect seismicity and deformation, and I think that is the most effective emphasis of the manuscript. The seismic analyses are sound and mostly well-described in the Methods and Supplemental Material - there is no need to redo any of the analyses. It's purely a question of how the results are interpreted/presented.

Discussion:

It's a shame the deployments were so short, because this limits the information content, but I think the first task of this paper should be to tell us what the results say about the nature of seismicity in a flip-flop detachment/smooth seafloor environment. I would expect the flip-flop style of detachment faulting, with its reversals of polarities, to impart a different set of fault structures to the lithosphere compared to normal detachment faulting. Figure 1b provides some insight into this. Compare, for example, the fault defined by E1-B1 with that defined by E2-B2. Flip-flopping should impart a unique fault structure, where older faults can be dismembered/disrupted by a newer, reversed polarity fault. What are the implications for seismicity associated with extension in these environments? Could this be why the authors find unusually high levels of activity in the hanging wall? They say that hanging wall seismicity may be a feature of flip-flop environments, but they don't say why. What is different about hanging wall structure and deformation in these environments that might lead to this behavior? It looks like footwalls can become hanging walls in these environments - what effects might that have? The work described in this manuscript is our first opportunity to know something about flip-flop detachment seismicity, and this needs to be discussed at more length.

The relationship between MOR seismicity depth and thermal environment has been the subject of numerous studies, but it is not simple (see Molnar - The Brittle-Plastic Transition, Earthquakes, Temperatures, and Strain Rates, JGR, 2020), and comparing seismicity depths between OBS experiments at different MOR sites is a tricky business. The most recent manuscript to take on the question is, I believe, Grevemeyer et al. 2019, who came to the 'opposite' conclusion - i.e., that

there is indeed a good relationship between seismicity depth and spreading rate. The Grevemeyer paper goes into more analytical detail than this manuscript, but could still be criticized as being too simplistic. For example, it states that the maximum seismicity depth at the Mid-Cayman Spreading Center is ~10 km, but inspection of Figure 1 in their paper reveals that this is only true directly beneath the Mt. Dent detachment, and that seismicity depth along the ridge axis away from the detachment reaches ~15 km. At face value this suggests that detachment faulting affects the depth of seismicity, either through mechanical means (e.g., fault structure) or thermal means. The Mt. Dent detachment, like the site of this manuscript, features a high-temperature hydrothermal field. Cann and Strens (1982) showed that in order to sustain high-temperature venting it is necessary to have a magma chamber cooling in the crust - the latent heat of crystallization is key. To my knowledge magma chambers have been found beneath every high-temperature MOR vent field where the data exists at sufficient resolution to make a determination, and I can only assume there is magma in the crust somewhere below the vent field at the SWIR 64°30'E site. Crustal magma injections have also been shown to be an intrinsic component of detachment faulting. We have to therefore consider the possibility that detachment faulting alters the relationship between seismicity depth and spreading rate at MORs, as well as the possibility that a crustal magma injection temporarily modifies the thermal environment beneath high-temperature vent fields. Both of these possibilities confound the ability to make a sweeping statement about the relationship between seismicity and thermal environment at this study site.

The authors have done a nice job with the data analysis, but no amount of analysis can overcome the fundamental limitations imposed by the OBS networks deployed in this study. The networks are small, both in aperture and number of instruments, and they are not in ideal configurations for earthquake analyses (e.g., one network consisted of crossing lines for active-source analyses). Most of the events were located outside the aperture of the network that detected them - in some cases well outside - and this, which results in all arrivals detected on the network having similar raypaths, combined with the relatively small number of arrivals available for hypocentral analysis, invariably leads to large uncertainties. This might not be obvious to the casual reader because the error bars shown in Figure 2 are relative, not absolute, uncertainties. This gives a false sense of depth accuracy. The absolute depth uncertainties are plotted in Figure 8 of the Supplemental Material. Here we can see that the true depth uncertainties for a given event are relatively large - on the order of 5 km in many cases. Are these 1-sigma uncertainties, 3-sigma uncertainties? This important details needs to be specified. In any case it is the absolute depth uncertainty that is relevant to any discussion of thermal environment and/or comparison with other studies, and these uncertainties should be plotted in Figure 2. The absolute depth uncertainties seem to be larger than the depth variations between sites that the authors try to interpret, and this is clearly a problem.

Supplementary Figure 8 also shows the event depth effects of using different velocity models, which are considerable. This underscores how difficult it can be to interpret differences in seismicity depth between two different experiments at a granular level, because they employ different seismic networks and use different velocity models. For this reason, differences of ~10-15% in maximum depth between any two seismicity studies (as in this manuscript) are likely not significant enough to interpret without a much more comprehensive comparative analysis.

Some comments on specific parts of the manuscript:

Abstract - The wording for the sentence beginning with 'As magma is the main heat carrier...' is awkward. Since it is perhaps the most important sentence in the Abstract it should be revised and clarified. I suggest beginning by stating the result - new microEQ data show..., and then stating why this is surprising - same thickness as more magmatic/volcanic segments.

No need to use the term 'new' in front of 'data' (twice in the Abstract).

The fact that the data were acquired with ocean bottom seismometers should be clearly stated in the Abstract.

I suggest restricting the citations you list for MOR seismicity studies to those that are directly relevant to your topics. As much as I love to be cited, the Pontbriand and Sohn (2014) has little/nothing to do with this paper, nor does the Tan et al (2016), etc. There is such a large number of papers on MOR seismicity that you need to restrict your list to ones that are directly relevant (e.g., detachment faults, ultra-slow ridges).

No need to use the term 'first' here ('...first estimates of the thickness...'). As with the Abstract, this type of adjective is implied and should be left to the reader to determine.

Line 36 - remove 'numerically'

Lines 74-81 - The observation of earthquakes clustering around the transition from smooth to volcanic seafloor is interesting, but I don't understand the interpretation. Serpentinization significantly weakens the rock, and likely localizes strain along serpentinized shear zones. Weakening the rock makes it more, not less, susceptible to brittle failure. However, serpentine exhibits velocity strengthening behavior at low sliding velocities, which has led to speculation that serpentinite shear zones may accommodate stable creep rather than discrete earthquakes. I don't think anyone really knows the answer as to how serpentinization affects fault behavior - it is still very much a topic of speculation. In any case, if magmatic sills intrude into a formation, this generates a localized stress perturbation, and that seems the most likely explanation for the observation, rather than the rheological effects of serpentinization.

Methods

Overall, the methods are sound, but, as discussed previously, one cannot overcome the issues associated with the seismic networks. There will be large uncertainties in the hypocentral estimates, and even more so in the focal mechanism estimates. The < 250° azimuthal gap used for estimating focal mechanisms is very large - even 180° would be a large gap. The problem is clear when the arrival polarities are shown on the beach balls in Figure 2a, but I suspect this detail will escape most readers. The authors say the fault plane uncertainty is < 35°, which seems small given the aforementioned azimuthal gaps. HASH gives out grades for the estimates - what sort of grades did these get? These should be listed in the Methods section. At what confidence level are the fault planes uncertain at 35°?

I still don't believe that one of the largest events (ML ~ 3) occurred at a depth of ~0 km, as shown in Supplemental Figures 6b & 7. The event in question is located ~3 network apertures (in the along-axis direction) away from the network, meaning the location, and depth especially are poorly constrained regardless of pick quality. It's purely a ray geometry issue. The authors reply indicated that they inspected this event and found nothing wrong, but I'm fairly certain there is a problem somewhere. It is physically implausible to have a large event at such a shallow depth, and events like this that locate near a grid boundary (seafloor), well away from the instrument network, are typically bad locations in my experience.

Referee #2

Review Summary: In this revised manuscript the authors describe results from an OBS microearthquake study at the SWIR 64°30'E - a site of flip-flop detachment faulting. To my knowledge, this is the first OBS seismicity experiment at a site of flip-flop detachments and smooth seafloor, such that this is the community's 'first look' at active faulting in this type of environment. The data are thus unique, and the results should be published. The authors have chosen to focus, though, on the depth of seismicity, and to use this to argue that the 'classic' relationship between seismicity depth and spreading rate does not hold for ultra-slow spreading ridges. I have two issues with this: 1) I don't think we can properly assess the relationship between earthquake depth and thermal environment without first having an understanding of how the flip-flop detachment environment influences seismicity and deformation, and 2) The manuscript only brushes the surface of the topic, and does not adequately account for uncertainties, both in the presented hypocenters, and in differences between OBS experiments conducted at different sites.

In my assessment the manuscript is thus not yet ready for publication. If the authors wish to keep the current emphasis on implications for thermal environment, a more thorough analysis is required, as I describe, below. I don't know if it is possible to adequately do this within the space constraints of Nature Comms & Environment. My recommendation, however, would be to focus on the seismicity of flip-flop detachment environments, as this is the truly unique aspect of the results. The authors have the opportunity to make the first assessment of how the somewhat bizarre mechanics of flip-flop detachment faulting affect seismicity and deformation, and I think that is the most effective emphasis of the manuscript. The seismic analyses are sound and mostly well-described in the Methods and Supplemental Material - there is no need to redo any of the analyses. It's purely a question of how the results are interpreted/presented.

We added a new figure (Fig. 3), emphasising the difference between flip-flop and corrugated detachment faults (see the section of 'Microseismicity at detachment systems').

We no longer discuss the relationship between the thermal regime and spreading rate but narrow our discussion to the relationship between the thermal regime and melt supply at an ultraslow spreading rate (see the section of 'Maximum depth of earthquakes and the axial thermal regime').

Discussion:

It's a shame the deployments were so short, because this limits the information content, but I think the first task of this paper should be to tell us what the results say about the nature of seismicity in a flip-flop detachment/smooth seafloor environment. I would expect the flip-flop style of detachment faulting, with its reversals of polarities, to impart a different set of fault structures to the lithosphere compared to normal detachment faulting. Figure 1b provides some insight into this. Compare, for example, the fault defined by E1-B1 with that defined by E2-B2. Flip-flopping should impart a unique fault structure, where older faults can be dismembered/disrupted by a newer, reversed polarity fault. What are the implications for seismicity associated with extension in these environments? Could this be why the authors find unusually high levels of activity in the hanging wall? They say that hanging wall seismicity may be a feature of flip-flop environments, but they don't say why. What is

different about hanging wall structure and deformation in these environments that might lead to this behavior? It looks like footwalls can become hanging walls in these environments - what effects might that have? The work described in this manuscript is our first opportunity to know something about flip-flop detachment seismicity, and this needs to be discussed at more length.

We have done so (See Fig. 3 and see the section of 'Microseismicity at detachment systems').

The relationship between MOR seismicity depth and thermal environment has been the subject of numerous studies, but it is not simple (see Molnar - The Brittle-Plastic Transition, Earthquakes, Temperatures, and Strain Rates, JGR, 2020), and comparing seismicity depths between OBS experiments at different MOR sites is a tricky business. The most recent manuscript to take on the question is, I believe, Grevemeyer et al. 2019, who came to the 'opposite' conclusion - i.e., that there is indeed a good relationship between seismicity depth and spreading rate. The Grevemeyer paper goes into more analytical detail than this manuscript, but could still be criticized as being too simplistic. For example, it states that the maximum seismicity depth at the Mid-Cayman Spreading Center is ~10 km, but inspection of Figure 1 in their paper reveals that this is only true directly beneath the Mt. Dent detachment, and that seismicity depth along the ridge axis away from the detachment reaches ~15 km. At face value this suggests that detachment faulting affects the depth of seismicity, either through mechanical means (e.g., fault structure) or thermal means. The Mt. Dent detachment, like the site of this manuscript, features a high-temperature hydrothermal field. Cann and Strens (1982) showed that in order to sustain high-temperature venting it is necessary to have a magma chamber cooling in the crust - the latent heat of crystallization is key. To my knowledge magma chambers have been found beneath every high-temperature MOR vent field where the data exists at sufficient resolution to make a determination, and I can only assume there is magma in the crust somewhere below the vent field at the SWIR 64°30'E site. Crustal magma injections have also been shown to be an intrinsic component of detachment faulting. We have to therefore consider the possibility that detachment faulting alters the relationship between seismicity depth and spreading rate at MORs, as well as the possibility that a crustal magma injection temporarily modifies the thermal environment beneath high-temperature vent fields. Both of these possibilities confound the ability to make a sweeping statement about the relationship between seismicity and thermal environment at this study site.

We narrow the discussion of the thermal regime to its relationship with melt supply only. The thermal regime of the flip-flop detachment fault at the SWIR 64°30'E has also been constrained in petrological aspects of exhumed serpentinites, suggesting a minimum depth of 18 km for the 800-1000°C isotherm (the geotherm and stress inferred in Figure 16 of Bickert et al., 2021@Gcube). This is consistent with our seismic proxy for the depth of the 650°C isotherm (15 km). Molnar 2020@JGR suggested that earthquakes may occur at 800°C or higher, which may correspond to high-stress plastic to semi-brittle deformation at the SWIR 64°30'E (Bickert et al., 2021@Gcube). However, such deformation may not be recorded by OBS.

For the Old City hydrothermal vent in our study area, it is not high but low temperature, like Lost City at the MAR (Cannat et al., 2019@ Goldschmidt Conference). This information was missed in the last version, which added in Fig. 1 caption.

The authors have done a nice job with the data analysis, but no amount of analysis can overcome the fundamental limitations imposed by the OBS networks deployed in this study. The networks are small, both in aperture and number of instruments, and they are not in ideal configurations for earthquake analyses (e.g., one network consisted of crossing lines for active-source analyses). Most of the events were located outside the aperture of the network that detected them - in some cases well outside - and this, which results in all arrivals detected on the network having similar raypaths, combined with the relatively small number of arrivals available for hypocentral analysis, invariably leads to large uncertainties. This might not be obvious to the casual reader because the error bars shown in Figure 2 are relative, not absolute, uncertainties. This gives a false sense of depth accuracy. The absolute depth uncertainties are plotted in Figure 8 of the Supplemental Material. Here we can see that the true depth uncertainties for a given event are relatively large - on the order of 5 km in many cases. Are these 1-sigma uncertainties, 3-sigma uncertainties? This important details needs to be specified. In any case it is the absolute depth uncertainty that is relevant to any discussion of thermal environment and/or comparison with other studies, and these uncertainties should be plotted in Figure 2. The absolute depth uncertainties seem to be larger than the depth variations between sites that the authors try to interpret, and this is clearly a problem.

Depth uncertainties in Supplementary Fig. 8 are 1-sigma uncertainties. This is now stated. We added the average absolute horizontal location uncertainty in Figs. 2a and 2c. Since we removed events with horizontal and depth errors of >5 km and RMS residual of >100 ms (Methods), the average error is ~3 km (1-sigma).

Supplementary Figure 8 also shows the event depth effects of using different velocity models, which are considerable. This underscores how difficult it can be to interpret differences in seismicity depth between two different experiments at a granular level, because they employ different seismic networks and use different velocity models. For this reason, differences of ~10-15% in maximum depth between any two seismicity studies (as in this manuscript) are likely not significant enough to interpret without a much more comprehensive comparative analysis.

The two different patterns of earthquake hypocenters shown in Supplementary Fig. 8 were located using two different velocity models: 1) from the nearly-amagmatic SWIR 64°30'E (study area; Momoh et al., 2017) and 2) from magmatically-robust SWIR 65-66°E (segment #8; Minshull et al., 2006), respectively. The second model, which is unsuitable for our study area and gives shallower EQ depths, indicates a maximum EQ depth of ~20 km (Supplementary Fig. 8b-2).

Some comments on specific parts of the manuscript:

Abstract - The wording for the sentence beginning with 'As magma is the main heat carrier...' is awkward. Since it is perhaps the most important sentence in the Abstract it should be revised and clarified. I suggest beginning by stating the result - new microEQ data show..., and then stating why this is surprising - same thickness as more magmatic/volcanic segments.
No need to use the term 'new' in front of 'data' (twice in the Abstract).
The fact that the data were acquired with ocean bottom seismometers should be clearly stated in the Abstract.

We revised the Abstract according to your comments.

I suggest restricting the citations you list for MOR seismicity studies to those that are directly relevant to your topics. As much as I love to be cited, the Pontbriand and Sohn (2014) has little/nothing to do with this paper, nor does the Tan et al (2016), etc. There is such a large number of papers on MOR seismicity that you need to restrict your list to ones that are directly relevant (e.g., detachment faults, ultra-slow ridges).

We removed these two references and other similar references. We added some references, e.g., Cann et al., (1997), Simão et al., (2020), and Zhao et al., (2012), which are relevant to detachment faults at slow and ultraslow spreading ridges.

No need to use the term 'first' here ('...first estimates of the thickness...'). As with the Abstract, this type of adjective is implied and should be left to the reader to determine.
Line 36 - remove 'numerically'

We removed these.

Lines 74-81 - The observation of earthquakes clustering around the transition from smooth to volcanic seafloor is interesting, but I don't understand the interpretation. Serpentinization significantly weakens the rock, and likely localizes strain along serpentinized shear zones. Weakening the rock makes it more, not less, susceptible to brittle failure. However, serpentine exhibits velocity strengthening behavior at low sliding velocities, which has led to speculation that serpentinite shear zones may accommodate stable creep rather than discrete earthquakes. I don't think anyone really knows the answer as to how serpentinization affects fault behavior - it is still very much a topic of speculation. In any case, if magmatic sills intrude into a formation, this generates a localized stress perturbation, and that seems the most likely explanation for the observation, rather than the rheological effects of serpentinization.

We now suggest that the lower seismicity beneath the smooth seafloor could be due do serpentinization creep, as suggested (Lines 66-71).

Methods

Overall, the methods are sound, but, as discussed previously, one cannot overcome the issues associated with the seismic networks. There will be large uncertainties in the hypocentral estimates, and even more so in the focal mechanism estimates. The < 250° azimuthal gap used for estimating focal mechanisms is very large - even 180° would be a large gap. The problem is clear when the arrival polarities are shown on the beach balls in Figure 2a, but I

suspect this detail will escape most readers. The authors say the fault plane uncertainty is < 35°, which seems small given the aforementioned azimuthal gaps. HASH gives out grades for the estimates - what sort of grades did these get? These should be listed in the Methods section. At what confidence level are the fault planes uncertain at 35°?

We added the HASH grades (in E) because of azimuthal gaps of all over 90°, and the confidence level is 95%. See Methods.

I still don't believe that one of the largest events (ML ~ 3) occurred at a depth of ~0 km, as shown in Supplemental Figures 6b & 7. The event in question is located ~3 network apertures (in the along-axis direction) away from the network, meaning the location, and depth especially are poorly constrained regardless of pick quality. It's purely a ray geometry issue. The authors reply indicated that they inspected this event and found nothing wrong, but I'm fairly certain there is a problem somewhere. It is physically implausible to have a large event at such a shallow depth, and events like this that locate near a grid boundary (seafloor), well away from the instrument network, are typically bad locations in my experience.

We agree with you that there may be a ray geometry issue in location. We added ray geometry criteria to our selection process, removing two large events, including this one, that are away from the network and mainly recorded by on-axis OBSs.

Reviewer comments, third round review:

Reviewer #2 (Remarks to the Author):

I appreciate the authors' efforts to revise the manuscript. Apart from some grammatical issues that I assume will be addressed by the copy editors, it is now suitable for publication.

I remain concerned about the small number of instruments used for the experiments, and the short length of the deployments. On the one hand, the small number of instruments make it difficult to obtain reliable hypocenter and focal mechanism estimates, and on the other, the short length of the deployments make it difficult to assess how representative the seismicity is for the unique geological setting.

Nevertheless, these are issues that can't be fixed, and the data and results are unique and should be published.

My only requested revision is for the authors to accurately write my last name in the deMartin et al (2006) citation.

Referee #2

Reviewer #2 (Remarks to the Author):

I appreciate the authors'; efforts to revise the manuscript. Apart from some grammatical issues that I assume will be addressed by the copy editors, it is now suitable for publication.

I remain concerned about the small number of instruments used for the experiments, and the short length of the deployments. On the one hand, the small number of instruments make it difficult to obtain reliable hypocenter and focal mechanism estimates, and on the other, the short length of the deployments make it difficult to assess how representative the seismicity is for the unique geological setting. Nevertheless, these are issues that can't be fixed, and the data and results are unique and should be published.

My only requested revision is for the authors to accurately write my last name in the deMartin et al (2006) citation.

It's corrected.
